# Antibiotic containing bone cement in prevention of hip and knee prosthetic joint infections: A systematic review and meta-analysis

**DOI:** 10.1016/j.jot.2020.04.005

**Published:** 2020-05-08

**Authors:** Sujeesh Sebastian, Yang Liu, Robin Christensen, Deepak Bushan Raina, Magnus Tägil, Lars Lidgren

**Affiliations:** a Lund University, Faculty of Medicine, Department of Clinical Sciences Lund, Orthopedics, Lund, Sweden; b Musculoskeletal Statistics Unit, The Parker Institute, Bispebjerg and Frederiksberg Hospital, Copenhagen & Research Unit of Rheumatology, Department of Clinical Research, University of Southern Denmark, Odense University Hospital, Denmark

**Keywords:** Antibiotic-loaded bone cement, Infection, Polymethyl methacrylate bone cement, Total knee arthroplasty, Total hip arthroplasty

## Abstract

**Background:**

Prosthetic joint infection (PJI) is the most serious total joint arthroplasty (TJA) complication despite several aseptic and antiseptic preventive measures. There is no clear evidence or even consensus, whether antibiotic-loaded bone cement (ALBC) should be used, in addition to systemic short-term routine antibiotic prophylaxis, to reduce the risk of PJI in primary TJA. We aimed to analyze the efficacy of ALBC for prevention of PJI in patients undergoing primary TJA.

**Methods:**

We searched systematically for randomized controlled trials (RCTs) in PubMed, Scopus, Embase, Web of Science and Cochrane library. Two reviewers independently screened potentially eligible studies according to predefined selection criteria and assessed the risk of bias using a modified version of the Cochrane risk of bias tool. PJI was prespecified as the primary outcome of interest. The meta-analyses were based on risk ratios using random-effects model per default. For the purpose of sensitivity, the corresponding fixed effects model odds ratios were calculated with the use of the Peto method as well. To evaluate a potential difference in effect sizes using different types (subgroups) of antibiotics used in bone cement, and at different follow-up periods, we performed stratified meta-analyses.

**Results:**

Thirty-seven studies were eligible for the systematic review and qualitative synthesis, and 9 trials (6507 total joint arthroplasties) were included in this meta-analysis. Overall ALBC significantly reduced the risk of PJI following primary TJAs (RRs, 0.36; 95% CIs, 0.16 to 0.80; *P* = 0.01) with a moderate degree of inconsistency (I^2^ = 47%). Based on stratified meta-analyses the use of gentamicin appeared to have a better effect (*P* = 0.0005) in the total hip arthroplasty. Pooled data of different antibiotics used in knee arthroplasties showed a significant association of cefuroxime (RRs, 0.08; 95% CIs, 0.01 to 0.63; *P* = 0.02). Further, ALBCs significantly reduced the PJI at one and two years of follow-up (*P* = 0.03 and *P* = 0.005 respectively).

**Conclusions:**

The evidence suggests that ALBCs are effective in reducing the PJI following primary TJA; i.e. between 20 and 84% reduced risk. However, the clear limitations of the available trial evidence highlight the need for joint-specific confirmatory trials, that will need to be designed as cluster-randomized trials of clinics in countries with well-functioning arthroplasty registries.

*The translational potential of this article:* This meta-analysis highlights the prophylactic potential of ALBCs in lowering the risk of infection following primary hip or knee arthroplasties but emphasizes the need for more recent confirmatory trials.

## Introduction

Antibiotic-loaded bone cement (ALBC) was introduced by Buchholz from the ENDO-Klinik in Germany in the 1970's for cemented hip revision of prosthetic joint infection (PJI) [1]. In 1979, the first reports of ABLC in combination with systemic antibiotics in primary arthroplasty was published [2,3]. For cemented revisions of mechanical failure, it is now established to use ABLC [4]. Also, in Europe and Australia, it is common practice to use ABLC in primary arthroplasties [[5], [6], [7], [8], [9], [10], [11]]. However, there are conflicting results from various prospective/retrospective studies and the use of ABLC in primary arthroplasty is still a subject of debate [[12], [13], [14], [15], [16]].

We aimed to evaluate the body of evidence linking ALBC with a reduction of PJI following primary arthroplasty based on randomized controlled trials (RCTs). Our primary objective was to determine the efficacy of polymethyl methacrylate (PMMA) with antibiotics on prevention of PJI in patients undergoing primary hip and knee arthroplasties. Stratified meta-analyses were performed to compare the efficacy of various antibiotics used in bone cement, and prophylactic potential of ALBCs at different time points following primary implantation.

## Methods

Study selection, assessment of eligibility criteria, data extraction, and statistical analyses were performed based on a predefined protocol in accordance with the current methodology guidelines [17].

### Data sources and search strategy

This study was conducted as a systematic review with subsequent meta-analysis reported based on the Preferred Reporting Items for Systematic Reviews and Meta-Analysis (PRISMA) guidelines [18]. We systematically searched for randomized controlled trials on ALBCs in primary arthroplasties available from the following databases PubMed (Medline), Scopus, Embase, Web of Science and Cochrane library.

Our search strategy was based on the PICO (patient, intervention, comparator and outcome) framework [19]: As medical subject headings or keywords we used P: (“hip arthroplasty/hip replacement” OR “knee arthroplasty/replacement”) AND I: (“ALBC” OR “PMMA with antibiotic” OR “bone cement” OR “gentamicin” OR “cefuroxime” OR “tobramycin” OR “vancomycin”) AND O: (“periprosthetic joint infection” OR “prosthetic joint infection” OR “prosthesis related infection”) combined with the use of the Cochrane highly sensitive search strategies for identifying randomized trials in the specific databases: (randomized controlled trial OR controlled clinical trial OR randomized OR placebo [tiab] OR clinical trials as topic [mesh:noexp] OR randomly [tiab] OR trial [ti] NOT (animals NOT humans)).

There was no language restriction for the articles. All articles published up to November 28, 2019 were screened. Reference lists of relevant studies, review articles and meta-analyses were searched manually for additional papers missed by the electronic search.

### Eligibility and selection criteria

Two reviewers (SS and YL) independently scrutinized potential papers for eligibility; in case of disagreement on study eligibility, senior author (LL) made the final decision. The studies were included if; 1) the study was on primary total hip arthroplasty (THA) and/or total knee arthroplasty (TKA) patients; 2) the control group included plain bone cement/systemic antibiotics and ALBC in the intervention group; and 3) the study appeared to be a randomized controlled trial. In order to be eligible for quantitative synthesis (i.e. meta-analysis) the trial had to report data on the incidence of PJI in patients with and without ALBCs. Studies were excluded if; 1) the details of the antibiotics used in bone cement were not available; and 2) full-text articles were not available.

### Data extraction and assessment of the methodological quality

To maintain accuracy, two authors (SS and YL) independently performed the data extraction and risk of bias assessments. The information extracted included the following study characteristics: author, year of publication, country, joint operated, number of arthroplasties included for evaluation, brand name of the ALBC used, name and amount of antibiotic used in ALBC, details of systemic antibiotic used, duration of follow-up, and incidence of PJI in both control and intervention group. The internal validity of the included studies was assessed using the Cochrane risk of bias tool [20].

### Statistical analysis

Serious Adverse Events like the PJI were collected as binary outcomes. Results in forest plots are relative risk (RR) estimates with 95% confidence intervals (95%CI). Thus, the overall association between treatment and PJI are reported as RRs (95%CI) combined using random-effects model based on the DerSimonian-Laird method [21]. To test for the robustness of our primary analyses, we also conducted *post hoc* sensitivity analysis. Since we expected PJI events to be rare, also the odds ratios (OR) estimates (with 95%CI) were calculated with the use of the Peto method corresponding to the use of a fixed effects model [22].

The Cochran Q-test and the *I*^2^ inconsistency statistic were used to examine statistical heterogeneity and explore treatment associations in stratified meta-analyses according to the following subgroups: (1) efficacy of various antibiotics used in ALBCs *vs* plain bone cement; (2) knee arthroplasty trials with ALBCs *vs.* other; (3) trials with longer vs shorter (≤12 months vs ≥ 24 months) follow-up.

Analyses were performed using Review Manager for meta-analyses (version 5.3 The Nordic Cochrane Centre, The Cochrane Collaboration).

## Results

### Search results

**Figure 1:**
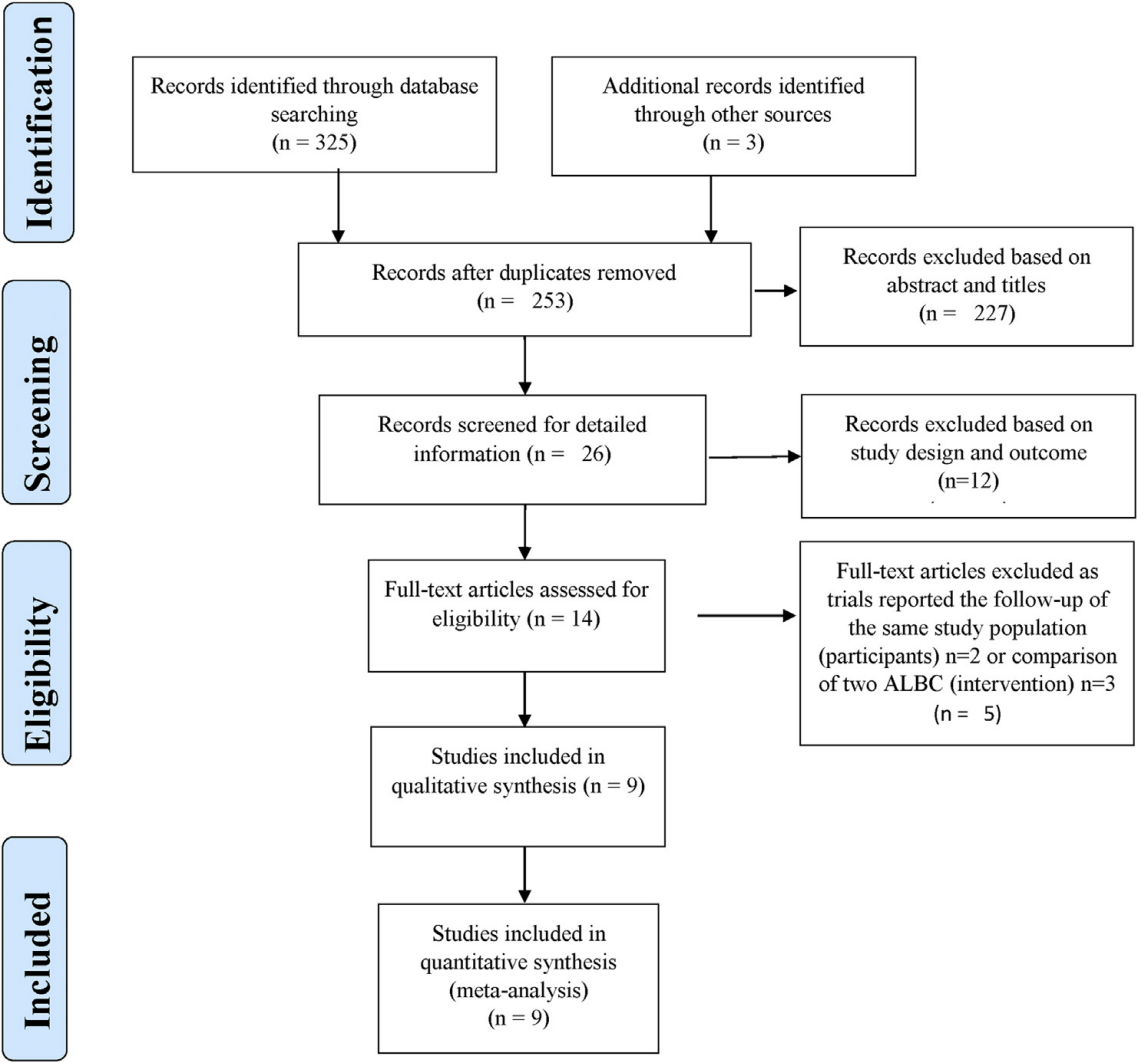
The Preferred Reporting Items for Systematic Reviews and Meta-Analyses (PRISMA) flow diagram of the included studies.

As illustrated in the flow diagram in Fig. 1, the database search yielded 325 articles and in addition three articles were included from other sources. After removal of duplicate articles 253 articles remained. After screening the title and abstracts of these, 227 articles were removed and detailed screening was performed for the remaining 26 articles. Among the 26 articles screened for detailed information, 9 articles were eligible for quantitative synthesis (i.e. meta-analysis); however, for practical reasons since one of these reported zero events in both groups this trial was not part of the combined estimate.

### Study characteristics

The detailed study characteristics of the nine RCTs included in this meta-analysis are given in Table 1. In total, 6507 TJAs were included (3296 in the intervention group and 3211 in control group) with sample sizes ranging from 78 to 2948 TJAs [2,3,12,13,[23], [24], [25], [26], [27]]. The majority of the studies were reported from European countries [2,3,12,13,23,24] and remaining from Asia [[25], [26], [27]]. Except one [3], all described the criteria used for the diagnosis of PJI and details are available as Appendix A.

**Table 1:** Characteristics of the included studies ALBCG: Antibiotic-loaded bone cement group; CG: Control group.

Study	Country	No of Joints (ALBCG:CG)	Operative Site	Cement Type	Antibiotic in cement	Systemic Antibiotic	Follow-up (Months)
Pfarr et al. (1979)	Germany	100:100	Hip	Palacos and Refobacin-Palacos	Gentamicin	Not mentioned	24
Wannske et al. (1979)	Germany	274:202	Hip	Palacos and Refobacin-Palacos	Gentamicin	Not mentioned	29
Josefsson et al. (1981)	Sweden	821:812	Hip	Palacos	Gentamicin (0.5 g)	Cephalotin and cephalexine, Cloxacillin, Cloxacillin and Probenecid, Dicloxacillin, Phenoxymethyl penicillin	12–24
McQueen et al. (1987)	United Kingdom	146:149	Hip and Knee	CMW	Cefuroxime (1.5 g)	Cefuroxime	3
McQueen et al. (1990)	United Kingdom	201:200	Hip and Knee	CMW	Cefuroxime (1.5 g)	Cefuroxime	24
Chiu et al. (2001)	China	41:37	Knee	Simplex P	Cefuroxime (2 g)	Cefazolin and gentamicin	50 (26–88)
Chiu et al. (2002)	China	178:162	Knee	Simplex P	Cefuroxime (2 g)	Cefazolin and gentamicin	49 (26–80)
Hinarejos et al. (2013)	Spain	1465:1483	Knee	Simplex P	Erythromycin (0.5 g) and colistin (three million units)	Cefazolin (or vancomycin if the patient had a beta-lactam allergy)	12
Huali et al. (2014)	China	70:66	Knee	Not mentioned	Vancomycin (1 g)	Cefazolin (or Azithromycin for patients allergic to cefazolin)	20.6 (4–24)

### Assessment of the internal validity and risk of bias in individual trials

**Figure 2:**
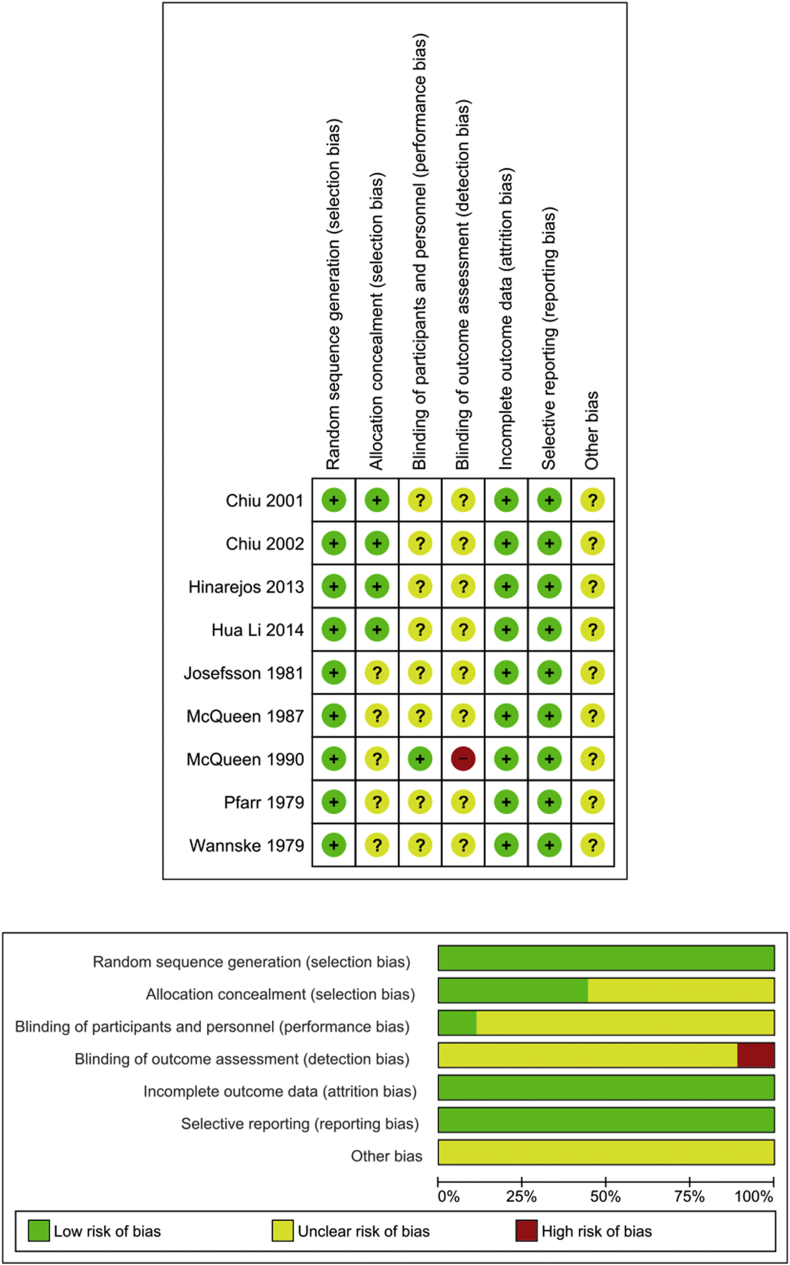
Risk of bias of included trials.

The quality assessment of each included study is illustrated in Fig. 2. In all included trials, the risk of random sequence generation was low. Allocation concealment was unclear in more than half of the studies [2,3,12,23,24]. Of the nine trials, performance bias was assessed as unclear in eight [2,3,12,13,23,[25], [26], [27]] and low in the remaining one trial [24]. Detection bias was unclear in eight trials [2,3,12,13,23,[25], [26], [27]] and one showed high-risk bias [24]. All trials showed a low-risk for attrition and reporting bias.

### Efficacy of ALBC in reducing the PJI

**Figure 3:**
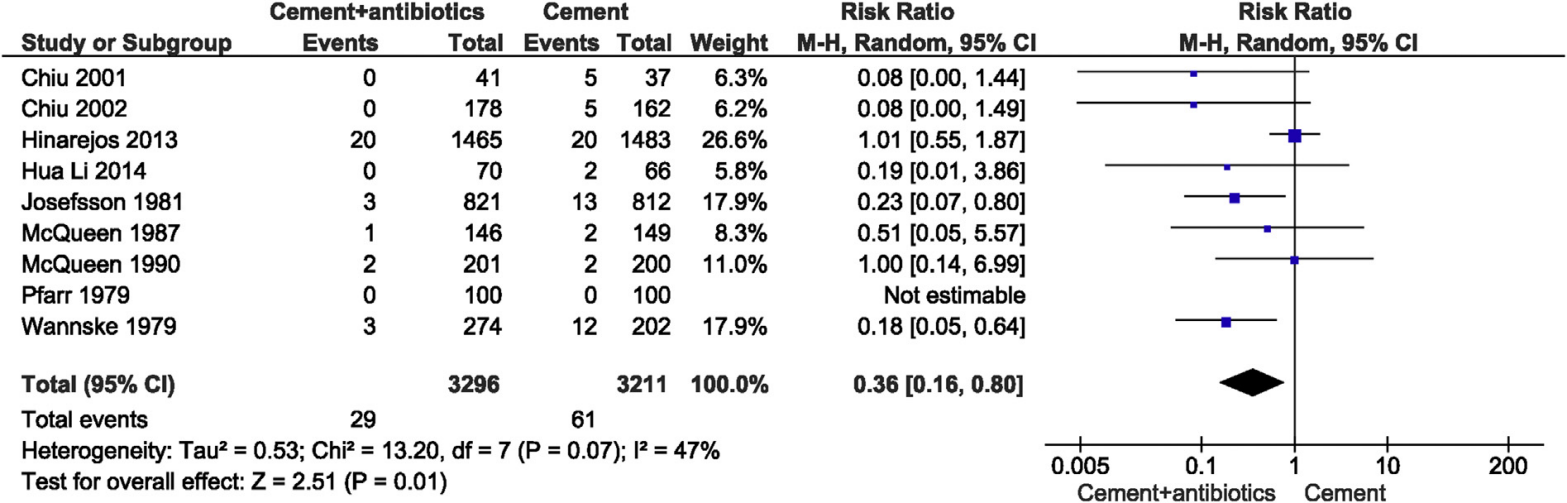
The RRs and 95% CIs for the incidence of prosthetic joint infection among patients treated with and without antibiotic-loaded bone cement.

The summary Risk Ratio for PJI was 0.36 in favor of the ALBC group (95%CI: 0.16 to 0.80; P = 0.03). As illustrated in Fig. 3 the overall pooled results on PJI's in the nine RCTs suggested that prophylactic use of ALBCs on average reduced the risk in primary TJA with 64% compared to control group with a moderate degree of heterogeneity (I^2^ = 47%).

These findings were considered statistically robust: In comparison with the primary analysis (Fig. 3) - when using the Peto OR - the pooled Peto OR (Appendix B) indicated reduced odds of having a PJI (Peto OR, 0.46 [95%CI: 0.30 to 0.69; P = 0.0002]).

### Stratified meta-analysis

#### (a) Efficacy of various antibiotics used in bone cements

**Figure 4:**
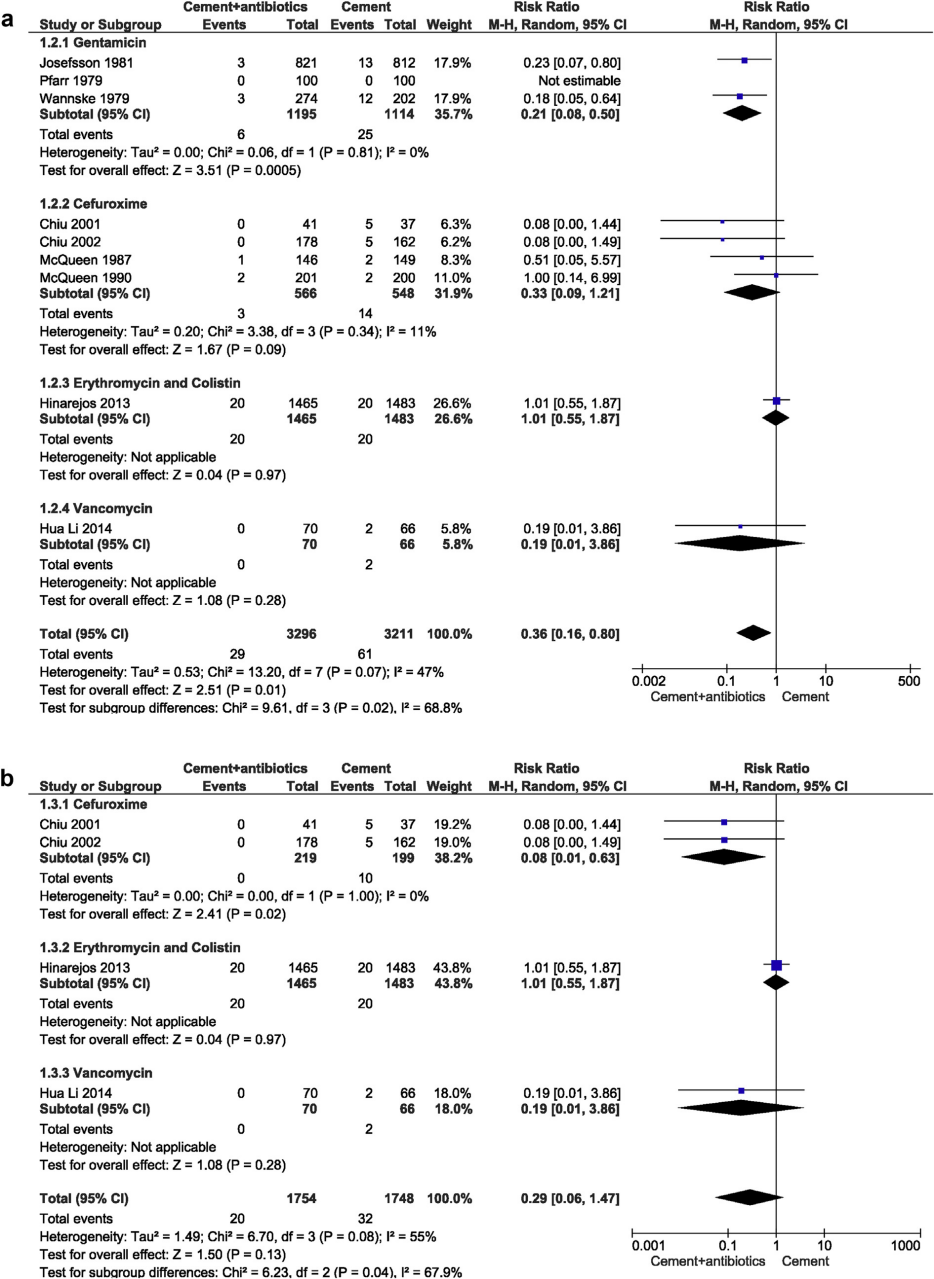

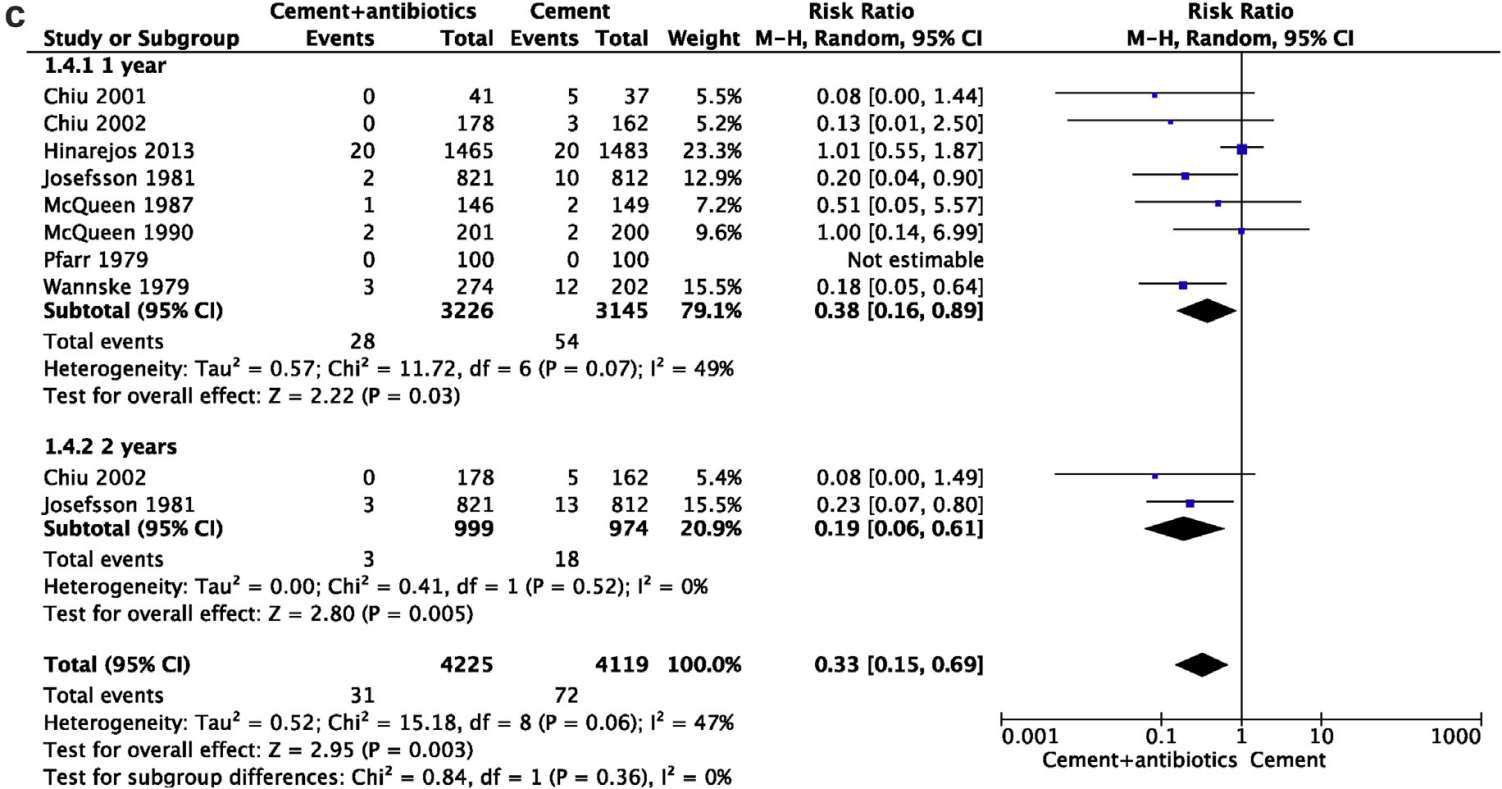
a: The RRs and 95% CIs for the incidence of prosthetic joint infection based on the antibiotics used in bone cement. b: The RRs and 95% CIs for the incidence of prosthetic joint infection based on the antibiotics used in knee arthroplasty bone cement. c: The RRs and 95% CIs for the incidence of prosthetic joint infection based on the follow-up time.

Fig. 4a presents the clinical risk according to the antibiotic used. Based on the antibiotic used in bone cement in each study, we analyzed the PJI rate among four different subgroups (gentamycin, cefuroxime, erythromycin & colistin and vancomycin). We found a statistically significant effect (*P* = 0.02), indicating that the four subgroups had a varying effect, and choice of antibiotic could be an effect modifier; possibly in favour of gentamicin. While a significant difference was found between antibiotic-impregnated PMMA and control treatments in the gentamycin (*P* = 0.0005) subgroup, no statistical significance was observed in the cefuroxime (*P* = 0.09), erythromycin & colistin (*P* = 0.97) or vancomycin subgroup (*P* = 0.28) (Fig. 4a).

#### (b) Efficacy of various antibiotics used in knee arthroplasty bone cements

We observed that among four RCTs in total knee arthroplasties, three types of antibiotics, namely cefuroxime, erythromycin & colistin and vancomycin were added to the bone cement [13,25,26]. Overall pooled results of these four RCTs showed no significant difference in PJI rate between ALBC and control patients following primary TKA (*P* = 0.13; Fig. 4b). However, in the cefuroxime subgroup, there was a statistically significant difference between antibiotic-impregnated PMMA and control arthroplasties (RRs, 0.08; 95% CIs, 0.01 to 0.63; *P* = 0.02) (Fig. 4b). In RCTs in total hip arthroplasties, only gentamicin was used [2,3,12] and thus no further analysis was performed.

#### (c) Efficacy of ALBCs at different follow-up periods

We further analyzed the PJI rate among the two different subgroups, with PJI developing within one-year and within two-years, based on the follow-up time in each study. The subtotal pooled results demonstrated that the use of ALBCs in primary TJA had a significant advantage over the control group in the prevention of PJI (*P* = 0.003) (Fig. 4c).

## Discussion

The clinical use of ALBCs in cemented revision arthroplasties is well established [28]. However, the routine use of ALBC in primary TJA, especially in knee arthroplasties is debated [[12], [13], [14], [15], [16]]. In this meta-analysis of nine currently available RCT's, it could be concluded that ALBCs seemed effective in reducing the incidence of PJI following primary cemented hip or knee arthroplasties compared to those receiving only plain PMMA cement in combination with systemic antibiotics.

### Comparison to the existing literature

Eight meta-analyses have previously evaluated the efficacy of ALBC during primary TJA (Hip = 2, Knee = 4, Hip and knee = 1, Hip, knee and shoulder = 1) in reducing PJI [[29], [30], [31], [32], [33], [34], [35], [36]]. Among these, a closest comparison could be made from Wang et al. study on both hip and knee RCTs in which a beneficial effect of ALBC was found for infection control in primary TJA [30]. However, in that meta-analysis, the study by Chiu et al. was excluded since patients with diabetes mellitus as the only co-morbidity were included in both the control and ALBC group [25]. Since no other outcome than PJI was analysed in the present study, we also excluded the study by Bohm et al. and added another new RCT reported by Huali et al. [27,37]. In a recent meta-analysis of ALBC used in primary knee arthroplasty, Kunutsor et al. reported no effect of ALBC in reducing the PJI, but non-randomized studies were included in their publication and there was only one single RCT comparing the effect of ALBC over plain bone cement [35].

The first RCTs that evaluated the efficacy of routine ALBC in primary arthroplasties were also the available RCTs on THA only [2,3,12]. These RCTs used the same antibiotic (0.5 g gentamicin) in bone cement and all reported that ALBC significantly reduced the PJI up to two-year follow-up. One study observed the same effect of ALBC at five-year but found no difference at longer follow-up [38,39]. A recent retrospective study from Spain not only reinforced the effectiveness but also showed the cost-benefit of ALBC in THA [14].

Contrary to the previous meta-analyses, we observed a significant effect of ALBC in reducing the PJI in primary TKA [[31], [32], [33], [34], [35]]. The potential reasons for the observed difference could be due to the exclusion of some of the RCTs from the analysis or in the previous meta-analyses, inclusion of prospective and retrospective studies or inclusion of RCTs with different outcomes other than infection as the primary outcome. When we further compared the data from retrospective and prospective studies, conflicting reports were found [14,15,[40], [41], [42], [43], [44], [45], [46], [47], [48]]. Of four registry-based studies, two of them reported an increased risk of infection in the ALBC group [41,44]. But the authors acknowledged the considerable variation in the type of antibiotic used by the surgeons and also the selective use of ALBC in high-risk patients. The retrospective cohort study by Bohm et al., using the Canadian Joint Replacement Registry and Hospital Morbidity Database reported a similar revision rate for infection in both ALBC and plain cement group [47]. Nevertheless, revision due to aseptic loosening was significantly higher in the plain cement group. A recent registry study by Jameson et al. analyzed 731 214 primary cemented TKAs using national joint registry data of England and Wales [49]. ALBC was associated with a significantly lower risk of revision for all causes: aseptic and infection. Highlighting an increased use in high-risk patients, it should still be noted that ALBC exhibited a significant effect in reducing the risk of revision. In a further recent population-based study, Chan et al. reported a decreased risk for early postoperative infection rate in ALBC group but with an increased risk for kidney injury [45]. Being the first to analyze such large data, the authors concluded to recommend the ALBC in primary TKA in high-risk patients without pre-existing renal diseases. There are limitations to that study, in particular the lack of information about the baseline renal status, type of antibiotic included in the ALBCs and cement brand used.

Gentamicin is one of the most studied antibiotic clinically [2,3,12]. The physical properties and antimicrobial spectrum of gentamicin favour its use in bone cement. The latest annual reports from major European and Australian arthroplasty registries showed extensive use of gentamicin in both primary hip and knee arthroplasties [[5], [6], [7], [8], [9], [10]]. The clinical use of cefuroxime in PMMA has not been explored in register studies [[23], [24], [25], [26]]. Recent reports revealed the variability of microbial aetiology in different geographical locations [50,51]. Also, the anatomic location shows differences regarding causative bacteria [52]. These factors should be considered for the selection of an optimal systemic antibiotic and for adding antibiotics to the bone cement.

Most non-randomized studies on routine use of ALBC in primary TKA used gentamicin or tobramycin [14,15,40,42,43,46,53]. No studies with tobramycin in bone cement have reported any efficacy in reducing the infection in primary TKA [15,40,42]. In the case of gentamicin, Sanz-Ruiz and co-workers reported a significant effect, whereas, Eveillard et al. found the effect to be close to the limit of significance (*P* = 0.07) [14,53]. From the subgroup analysis of ALBCs used in knee arthroplasties, we found a significant effect of cefuroxime. However, none of the previous non-randomized studies reported the use of cefuroxime impregnated bone cement in primary TKA [14,15,40,42,43,46,53]. We strongly recommend that RCT studies, in the future investigate the potential of cefuroxime in ABLC in primary TKA.

The use of ALBCs in primary TJAs had an advantage over the control group in the prevention of PJI with a follow-up up to two-years. Future studies with longer follow-up periods are required to analyze the long-term prophylactic effect of the ALBCs.

Limitations: There are clear limitations to this study. First, the majority of the nine RCTs included in this meta-analysis were conducted more than 15 years ago, questioning the strength of the data with current standards of joint arthroplasties [2,3,12,[23], [24], [25], [26]]. Though there was a scarcity of RCTs, we strictly excluded all non-randomized studies. Secondly, the diagnostic criteria used for PJI in the individual studies included in this study were different. This may have affected the results of the meta-analysis. Third, the authors acknowledge the performance bias from the heterogenicity of the included studies. Fourth, though the subgroup analysis revealed the significance of gentamicin and cefuroxime in the reduction of infection, analysis of brand of cement as a possible confounder for the obtained result was not done in the present study. Finally, since this meta-analysis was concentrated only on studies with PJI as the primary outcome, the role of ALBCs in aseptic loosening, increased costs and antimicrobial resistance was not evaluated.

### Clinical implications and future directions

In light of the limitations of the small-sized available RCTs, there is a demand of well-planned register studies using cluster-randomization of clinical units taking into account the various confounders. Along with the patient-related factors, the effect of following factors should be included in the outcome of such studies: type and dose of antibiotic used in cement, cement brand, cement mixing and delivery systems, operative site, type and timing of parallel systemic antibiotic administration, anaesthetic procedure, implant and surgical approach, surgery time, duration of follow-up, type of hospital etc. The outcome indicators should not be limited only to PJI or aseptic loosening but also especially in case of a novel ABLC follow the emergence of antibiotic resistance, allergic complications, risk of renal failure and cost-benefit.

## Conclusions

Our current literature study on the routine use of ALBCs in preventing PJIs in primary total hip and knee arthroplasty showed evidence of efficacy in combination with systemic prophylaxis. However, the lack of recently conducted high-quality RCTs highlights the need for larger, rigorously conducted RCTs. Due to the size and cost of such studies, this might be feasible only by cluster randomization. The national registries in countries where a majority of hospitals participate are probably the best suited to carry out such studies.

## Funding and acknowledgements

We deeply acknowledge 10.13039/501100004359Swedish Research Council (Grant number: VR, 2015–0671) for financial support. Professor Christensen would like to acknowledge that the 10.13039/100014547Parker Institute, Bispebjerg and Frederiksberg Hospital is supported by a core grant from the 10.13039/100001275Oak Foundation (OCAY-18-774-OFIL).

## Declaration of Competing Interest

LL is a board member of Bone Support AB, Lund, Sweden and Ortho Cell, Australia. All other authors have no conflicts of interest to disclose in relation to this article.

## Appendix A Supplementary data

Multimedia component 1 Multimedia component 1

Multimedia component 2 The Peto ORs and 95% CIs for the incidence of prosthetic joint infection among patients treated with and without antibiotic-loaded bone cement Multimedia component 2

## References

[1] Buchholz H.W., Engelbrecht H. (1970). Depot effects of various antibiotics mixed with Palacos resins. Chirurg.

[2] Pfarr B., Burri C. (1979). Prospective study on the effect of gentamycin-Palacos in 200 total hip prostheses. Aktuelle Proble Chir Orthopadie.

[3] Wannske M., Tscherne H. (1979). Results of prophylactic use of Refobacin-Palacos in implantation of endoprostheses of the hip joint in Hannover. Aktuelle Proble Chir Orthopadie.

[4] Bini S.A., Chan P.H., Inacio M.C., Paxton E.W., Khatod M. (2016). Antibiotic cement was associated with half the risk of re-revision in 1,154 aseptic revision total knee arthroplasties. Acta Orthop.

[5] Sundberg M., Lindgren L., W-Dahl A., Robertsson O. (2019). Annual report.

[6] Kärrholm J., Rogmark C., Nauclér E., Vinblad J., Mohaddes M., Rolfson O. (2018). Annual report. Sweden.

[7] Australian Orthopaedic Association National Joint Replacement Registry (2019). Supplementary report- cement in hip and knee arthroplasty. https://aoanjrr.sahmri.com/documents/10180/671402/Cement+in+Hip+%26+Knee+Arthroplasty.

[8] National Joint Registry Annual report 2019, Hemel Hempstead, England. https://reports.njrcentre.org.uk/.

[9] Norwegian National Advisory Unit on Arthroplasty and Hip Fractures Annual report 2019. http://nrlweb.ihelse.net/eng/Rapporter/Report2019_english.pdf.

[10] Dutch Arthroplasty Register Online LROI annual report 2019. https://www.lroi-rapportage.nl/media/pdf/PDF%20Online%20LROI%20annual%20report%202019.

[11] Berberich C., Sanz-Ruiz P. (2019). Risk assessment of antibiotic resistance development by antibiotic-loaded bone cements: is it a clinical concern?. EFORT Open Rev.

[12] Josefsson G., Lindberg L., Wiklander B. (1981). Systemic antibiotics and gentamicin-containing bone cement in the prophylaxis of postoperative infections in total hip arthroplasty. Clin Orthop Relat Res.

[13] Hinarejos P., Guirro P., Leal J., Montserrat F., Pelfort X., Sorli M.L. (2013). The use of erythromycin and colistin-loaded cement in total knee arthroplasty does not reduce the incidence of infection: a prospective randomized study in 3000 knees. J Bone Joint Surg Am.

[14] Sanz-Ruiz P., Matas-Diez J.A., Sanchez-Somolinos M., Villanueva-Martinez M., Vaquero-Martin J. (2017). Is the commercial antibiotic-loaded bone cement useful in prophylaxis and cost saving after knee and hip joint arthroplasty? The transatlantic paradox. J Arthroplasty.

[15] Anis H.K., Sodhi N., Faour M., Klika A.K., Mont M.A., Barsoum W.K. (2019). Effect of antibiotic-impregnated bone cement in primary total knee arthroplasty. J Arthroplasty.

[16] Fillingham Y., Greenwald A.S., Greiner J., Oshkukov S., Parsa A., Porteous A. (2019). Hip and knee section, prevention, local antimicrobials: proceedings of international consensus on orthopedic infections. J Arthroplasty.

[17] Shamseer L., Moher D., Clarke M., Ghersi D., Liberati A., Petticrew M. (2015). Preferred reporting items for systematic review and meta-analysis protocols (PRISMA-P) 2015. Elob and Explanation.

[18] Moher D., Liberati A., Tetzlaff J., Altman D.G. (2009). Preferred reporting items for systematic reviews and meta-analyses: the PRISMA statement. Bmj.

[19] Schardt C., Adams M.B., Owens T., Keitz S., Fontelo P. (2007). Utilization of the PICO framework to improve searching PubMed for clinical questions. BMC Med Inf Decis Making.

[20] Higgins J.P., Altman D.G., Gotzsche P.C., Juni P., Moher D., Oxman A.D. (2011). The Cochrane Collaboration's tool for assessing risk of bias in randomised trials. Bmj.

[21] DerSimonian R., Laird N. (1986). Meta-analysis in clinical trials. Contr Clin Trials.

[22] Bradburn M.J., Deeks J.J., Berlin J.A., Russell Localio A. (2007). Much ado about nothing: a comparison of the performance of meta-analytical methods with rare events. Stat Med.

[23] McQueen M., Littlejohn A., Hughes S.P. (1987). A comparison of systemic cefuroxime and cefuroxime loaded bone cement in the prevention of early infection after total joint replacement. Int Orthop.

[24] McQueen M.M., Hughes S.P., May P., Verity L. (1990). Cefuroxime in total joint arthroplasty. Intravenous or in bone cement. J Arthroplasty.

[25] Chiu F.Y., Lin C.F., Chen C.M., Lo W.H., Chaung T.Y. (2001). Cefuroxime-impregnated cement at primary total knee arthroplasty in diabetes mellitus. A prospective, randomised study. J Bone Joint Surg Br.

[26] Chiu F.Y., Chen C.M., Lin C.F., Lo W.H. (2002). Cefuroxime-impregnated cement in primary total knee arthroplasty: a prospective, randomized study of three hundred and forty knees. J Bone Joint Surg Am.

[27] Hua Li Y.W., Tong Lei (2014). Application of antibiotic loaded bone cement in total knee arthroplasty. J Med Theor Prac.

[28] Cui Q., Mihalko W.M., Shields J.S., Ries M., Saleh K.J. (2007). Antibiotic-impregnated cement spacers for the treatment of infection associated with total hip or knee arthroplasty. J Bone Joint Surg Am.

[29] Parvizi J., Saleh K.J., Ragland P.S., Pour A.E., Mont M.A. (2008). Efficacy of antibiotic-impregnated cement in total hip replacement. Acta Orthop.

[30] Wang J., Zhu C., Cheng T., Peng X., Zhang W., Qin H. (2013). A systematic review and meta-analysis of antibiotic-impregnated bone cement use in primary total hip or knee arthroplasty. PloS One.

[31] Yi Z., Bin S., Jing Y., Zongke Z., Pengde K., Fuxing P. (2014). No decreased infection rate when using antibiotic-impregnated cement in primary total joint arthroplasty. Orthopedics.

[32] Zhou Y., Li L., Zhou Q., Yuan S., Wu Y., Zhao H. (2015). Lack of efficacy of prophylactic application of antibiotic-loaded bone cement for prevention of infection in primary total knee arthroplasty: results of a meta-analysis. Surg Infect.

[33] Kleppel D., Stirton J., Liu J.Y., Ebraheim N.A. (2017). Antibiotic bone cement's effect on infection rates in primary and revision total knee arthroplasties. World J Orthoped.

[34] King J.D., Hamilton D.H., Jacobs C.A., Duncan S.T. (2018). The Hidden cost of commercial antibiotic-loaded bone cement: a systematic review of clinical results and cost implications following total knee arthroplasty. J Arthroplasty.

[35] Kunutsor S.K., Wylde V., Whitehouse M.R., Beswick A.D., Lenguerrand E., Blom A.W. (2019). Influence of fixation methods on prosthetic joint infection following primary total knee replacement: meta-analysis of observational cohort and randomised intervention studies. J Clin Med.

[36] Kunutsor S.K., Beswick A.D., Whitehouse M.R., Blom A.W., Lenguerrand E. (2019). Implant fixation and risk of prosthetic joint infection following primary total hip replacement: meta-analysis of observational cohort and randomised intervention studies. J Clin Med.

[37] Bohm E., Petrak M., Gascoyne T., Turgeon T. (2012). The effect of adding tobramycin to Simplex P cement on femoral stem micromotion as measured by radiostereometric analysis: a 2-year randomized controlled trial. Acta Orthop.

[38] Josefsson G., Gudmundsson G., Kolmert L., Wijkstrom S. (1990). Prophylaxis with systemic antibiotics versus gentamicin bone cement in total hip arthroplasty. A five-year survey of 1688 hips. Clin Orthop Relat Res.

[39] Josefsson G., Kolmert L. (1993). Prophylaxis with systematic antibiotics versus gentamicin bone cement in total hip arthroplasty. A ten-year survey of 1,688 hips. Clin Orthop Relat Res.

[40] Gandhi R., Razak F., Pathy R., Davey J.R., Syed K., Mahomed N.N. (2009). Antibiotic bone cement and the incidence of deep infection after total knee arthroplasty. J Arthroplasty.

[41] Namba R.S., Chen Y., Paxton E.W., Slipchenko T., Fithian D.C. (2009). Outcomes of routine use of antibiotic-loaded cement in primary total knee arthroplasty. J Arthroplasty.

[42] Qadir R., Sidhu S., Ochsner J.L., Meyer M.S., Chimento G.F. (2014). Risk stratified usage of antibiotic-loaded bone cement for primary total knee arthroplasty: short term infection outcomes with a standardized cement protocol. J Arthroplasty.

[43] Wang H., Qiu G.X., Lin J., Jin J., Qian W.W., Weng X.S. (2015). Antibiotic bone cement cannot reduce deep infection after primary total knee arthroplasty. Orthopedics.

[44] Tayton E.R., Frampton C., Hooper G.J., Young S.W. (2016). The impact of patient and surgical factors on the rate of infection after primary total knee arthroplasty: an analysis of 64,566 joints from the New Zealand Joint Registry. Bone Joint Lett J.

[45] Chan J.J., Robinson J., Poeran J., Huang H.H., Moucha C.S., Chen D.D. (2019). Antibiotic-loaded bone cement in primary total knee arthroplasty: utilization patterns and impact on complications using a national database. J Arthroplasty.

[46] Turhan S. (2019). Does the use of antibiotic-loaded bone cement have an effect on deep infection in primary total knee arthroplasty practice?. Surg Infect.

[47] Bohm E., Zhu N., Gu J., de Guia N., Linton C., Anderson T. (2014). Does adding antibiotics to cement reduce the need for early revision in total knee arthroplasty?. Clin Orthop Relat Res.

[48] Yayac M., Rondon A.J., Tan T.L., Levy H., Parvizi J., Courtney P.M. (2019). The economics of antibiotic cement in total knee arthroplasty: added cost with No reduction in infection rates. J Arthroplasty.

[49] Jameson S.S., Asaad A., Diament M., Kasim A., Bigirumurame T., Baker P. (2019). Antibiotic-loaded bone cement is associated with a lower risk of revision following primary cemented total knee arthroplasty: an analysis of 731,214 cases using National Joint Registry data. Bone Joint Lett J.

[50] Benito N., Franco M., Ribera A., Soriano A., Rodriguez-Pardo D., Sorli L. (2016). Time trends in the aetiology of prosthetic joint infections: a multicentre cohort study. Clin Microbiol Infect.

[51] Sebastian S., Malhotra R., Sreenivas V., Kapil A., Chaudhry R., Dhawan B. (2019). A clinico-microbiological study of prosthetic joint infections in an Indian tertiary care hospital: role of universal 16S rRNA gene polymerase chain reaction and sequencing in diagnosis. Indian J Orthop.

[52] Tande A.J., Patel R. (2014). Prosthetic joint infection. Clin Microbiol Rev.

[53] Eveillard M., Mertl P., Tramier B., Eb F. (2003). Effectiveness of gentamicin-impregnated cement in the prevention of deep wound infection after primary total knee arthroplasty. Infect Control Hosp Epidemiol.

