# Supplementary material for: Antibiotic containing bone cement in prevention of hip and knee prosthetic joint infections: A systematic review and meta-analysis
Source: J Orthop Translat. 2020 May 8;23:53–60. doi: 10.1016/j.jot.2020.04.005 (PMC7256060; doi:10.1016/j.jot.2020.04.005)
Supplement: Multimedia component 1 [file mmc1.docx]

**Appendix A:** Definition of prosthetic joint infection used and the incidence of prosthetic joint infection in the eligible randomized controlled trials

| **Study** | **Definition used for PJI** | **PJI incidence, n (%)** | |
| --- | --- | --- | --- |
|  |  | **ALBCG** | **CG** |
| Pfarr *et al* (1979) | Based on the following criteria: pain, elevation of ESR, leucocytosis, and fistula. | 0(0%) | 0(0%) |
| Wannske *et al* (1979) | Not mentioned. | 3(1.1%) | 12(5.9%) |
| Josefsson *et al* (1981) | Based on the following three criteria: pain, elevated ESR (more than 35 mm per hour) and progressive radiographic resorption of bone stock. | 3(0.4%) | 13(1.6%) |
| McQueen *et al* (1987) | Infection extending beneath the deep fascia, with persistent wound discharge or joint pain and positive or negative cultures from deep tissues. | 1(0.7%) | 2(1.3%) |
| McQueen *et al* (1990) | Infection extending deep to the deep fascia, with persistent wound discharge or joint pain, positive or negative cultures from deep tissues and delay in wound healing. | 2(1%) | 2(1%) |
| Chiu *et al* (2001) | Infection extending deep to the deep fascia, with persistent wound discharge or joint pain, ESR and C-reactive protein (CRP) levels, positive or negative cultures from joint fluid/deep tissues and delay in wound healing. | 0 | 5(13.5%) |
| Chiu *et al* (2002) | Infection extending deep to the deep fascia, with persistent wound discharge or joint pain, ESR and C-reactive protein (CRP) levels, positive or negative cultures from joint fluid/deep tissues and delay in wound healing. | 0 | 5(3.1%) |
| Hinarejos *et al* (2013) | Criteria of the surgical wound infection by Centers for Disease Control and Prevention. | 20(1.35%) | 20(1.37%) |
| Huali *et al* (2014) | Based on the following two criteria: ESR and C-reactive protein (CRP) levels, positive or negative intraoperative cultures. | 0 | 2 (3%) |

ALBCG: Antibiotic-loaded bone cement group; CG: Control group

**Appendix C**: PRISMA Checklist

| **Section/topic** | **#** | **Checklist item** | **Reported on page #** |
| --- | --- | --- | --- |
| **TITLE** | | |  |
| Title | 1 | Identify the report as a systematic review, meta-analysis, or both. | 1 |
| **ABSTRACT** | | |  |
| Structured summary | 2 | Provide a structured summary including, as applicable: background; objectives; data sources; study eligibility criteria, participants, and interventions; study appraisal and synthesis methods; results; limitations; conclusions and implications of key findings; systematic review registration number. | 1 |
| **INTRODUCTION** | | |  |
| Rationale | 3 | Describe the rationale for the review in the context of what is already known. | 1 |
| Objectives | 4 | Provide an explicit statement of questions being addressed with reference to participants, interventions, comparisons, outcomes, and study design (PICOS). | 2 |
| **METHODS** | | |  |
| Protocol and registration | 5 | Indicate if a review protocol exists, if and where it can be accessed (e.g., Web address), and, if available, provide registration information including registration number. | 2  Not registered |
| Eligibility criteria | 6 | Specify study characteristics (e.g., PICOS, length of follow-up) and report characteristics (e.g., years considered, language, publication status) used as criteria for eligibility, giving rationale. | 2 |
| Information sources | 7 | Describe all information sources (e.g., databases with dates of coverage, contact with study authors to identify additional studies) in the search and date last searched. | 2 |
| Search | 8 | Present full electronic search strategy for at least one database, including any limits used, such that it could be repeated. | 2 |
| Study selection | 9 | State the process for selecting studies (i.e., screening, eligibility, included in systematic review, and, if applicable, included in the meta-analysis). | 2 |
| Data collection process | 10 | Describe method of data extraction from reports (e.g., piloted forms, independently, in duplicate) and any processes for obtaining and confirming data from investigators. | 2 |
| Data items | 11 | List and define all variables for which data were sought (e.g., PICOS, funding sources) and any assumptions and simplifications made. | 2 |
| Risk of bias in individual studies | 12 | Describe methods used for assessing risk of bias of individual studies (including specification of whether this was done at the study or outcome level), and how this information is to be used in any data synthesis. | 2 |
| Summary measures | 13 | State the principal summary measures (e.g., risk ratio, difference in means). | 2 |
| Synthesis of results | 14 | Describe the methods of handling data and combining results of studies, if done, including measures of consistency (e.g., I^2^) for each meta-analysis. | 2 |

| Risk of bias across studies | 15 | Specify any assessment of risk of bias that may affect the cumulative evidence (e.g., publication bias, selective reporting within studies). | 2 |
| --- | --- | --- | --- |
| Additional analyses | 16 | Describe methods of additional analyses (e.g., sensitivity or subgroup analyses, meta-regression), if done, indicating which were pre-specified. | 2 |
| **RESULTS** | | |  |
| Study selection | 17 | Give numbers of studies screened, assessed for eligibility, and included in the review, with reasons for exclusions at each stage, ideally with a flow diagram. | 2  Figure 1 |
| Study characteristics | 18 | For each study, present characteristics for which data were extracted (e.g., study size, PICOS, follow-up period) and provide the citations. | 2  Table 1  Appendix A |
| Risk of bias within studies | 19 | Present data on risk of bias of each study and, if available, any outcome level assessment (see item 12). | 2  Figure 2 |
| Results of individual studies | 20 | For all outcomes considered (benefits or harms), present, for each study: (a) simple summary data for each intervention group (b) effect estimates and confidence intervals, ideally with a forest plot. | 2-5  Table 1  Figure 3-4c  Appendix A-B |
| Synthesis of results | 21 | Present results of each meta-analysis done, including confidence intervals and measures of consistency. | 2-5  Table 1  Figure 3-4c  Appendix A-B |
| Risk of bias across studies | 22 | Present results of any assessment of risk of bias across studies (see Item 15). | 2  Figure 2 |
| Additional analysis | 23 | Give results of additional analyses, if done (e.g., sensitivity or subgroup analyses, meta-regression [see Item 16]). | 2-5  Figure 4a-4c |
| **DISCUSSION** | | |  |
| Summary of evidence | 24 | Summarize the main findings including the strength of evidence for each main outcome; consider their relevance to key groups (e.g., healthcare providers, users, and policy makers). | 5 |
| Limitations | 25 | Discuss limitations at study and outcome level (e.g., risk of bias), and at review-level (e.g., incomplete retrieval of identified research, reporting bias). | 5,7 |
| Conclusions | 26 | Provide a general interpretation of the results in the context of other evidence, and implications for future research. | 7 |
| **FUNDING** | | |  |
| Funding | 27 | Describe sources of funding for the systematic review and other support (e.g., supply of data); role of funders for the systematic review. | Attached |
