# Supplementary figures and images for: Antibiotic containing bone cement in prevention of hip and knee prosthetic joint infections: A systematic review and meta-analysis
Source: J Orthop Translat. 2020 May 8;23:53–60. doi: 10.1016/j.jot.2020.04.005 (PMC7256060; doi:10.1016/j.jot.2020.04.005)

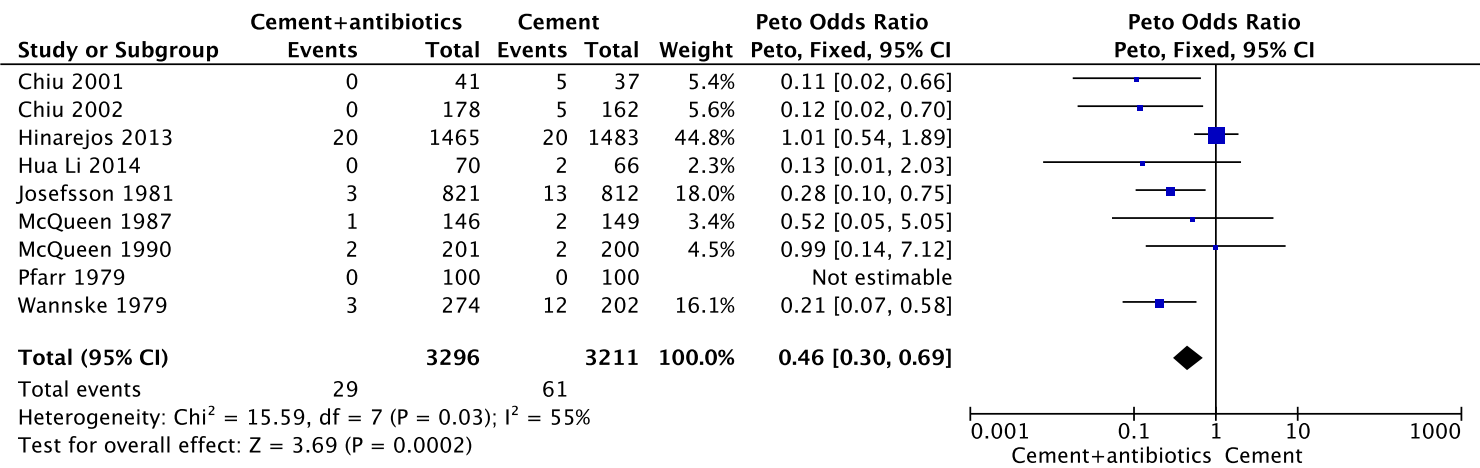

Supplement: Multimedia component 2 — The Peto ORs and 95% CIs for the incidence of prosthetic joint infection among patients treated with and without antibiotic-loaded bone cement [file mmc2.pdf]
